# Electron Spin Resonance Probe Incorporation into Bioinks Permits Longitudinal Oxygen Imaging of Bioprinted Constructs

**DOI:** 10.1007/s11307-023-01871-0

**Published:** 2023-12-01

**Authors:** Sajad Sarvari, Duncan McGee, Ryan O’Connell, Oxana Tseytlin, Andrey A. Bobko, Mark Tseytlin

**Affiliations:** 1 Department of Pharmaceutical Sciences, School of Pharmacy, West Virginia University, Morgantown, WV USA; 2 In Vivo Multifunctional Magnetic Resonance Center at Robert C. Byrd Health Sciences Center, West Virginia University, Morgantown, WV USA; 3 Department of Chemical and Biomedical Engineering, West Virginia University, Morgantown, WV USA; 4 Department of Biochemistry and Molecular Medicine, West Virginia University, Morgantown, WV USA; 5 West Virginia University Cancer Institute, Morgantown, WV USA

**Keywords:** Bioprinting, Oximetry, EPR Imaging, Rapid Scan EPR, GelMA, LiNc-BuO, Photo-Crosslinking, Hypoxia, Microfluidics, Bioink

## Abstract

**Purpose:**

Bioprinting is an additive manufacturing technology analogous to 3D printing. Instead of plastic or resin, cell-laden hydrogels are used to produce a construct of the intended biological structure. Over time, cells transform this construct into a functioning tissue or organ. The process of printing followed by tissue maturation is referred to as 4D bioprinting. The fourth dimension is temporal. Failure to provide living cells with sufficient amounts of oxygen at any point along the developmental timeline may jeopardize the bioprinting goals. Even transient hypoxia may alter cells' differentiation and proliferation or trigger apoptosis. Electron paramagnetic resonance (EPR) imaging modality is proposed to permit 4D monitoring of oxygen within bioprinted structures.

**Procedures:**

Lithium octa-n-butoxy-phthalocyanine (LiNc-BuO) probes have been introduced into gelatin methacrylate (GelMA) bioink. GelMA is a cross-linkable hydrogel, and LiNc-BuO is an oxygen-sensitive compound that permits longitudinal oximetric measurements. The effects of the oxygen probe on printability have been evaluated. A digital light processing (DLP) bioprinter was built in the laboratory. Bioprinting protocols have been developed that consider the optical properties of the GelMA/LiNc-BuO composites. Acellular and cell-laden constructs have been printed and imaged. The post-printing effect of residual photoinitiator on oxygen depletion has been investigated.

**Results:**

Models have been successfully printed using a lab-built bioprinter. Rapid scan EPR images reflective of the expected oxygen concentration levels have been acquired. An unreported problem of oxygen depletion in bioprinted constructs by the residual photoinitiator has been documented. EPR imaging is proposed as a control method for its removal. The oxygen consumption rates by HEK293T cells within a bioprinted cylinder have been imaged and quantified.

**Conclusions:**

The feasibility of the cointegration of 4D EPR imaging and 4D bioprinting has been demonstrated. The proof-of-concept experiments, which were conducted using oxygen probes loaded into GelMA, lay the foundation for a broad range of applications, such as bioprinting with many types of bioinks loaded with diverse varieties of molecular spin probes.

## Introduction

Bioprinting is an additive manufacturing technology intended for the engineering of functional human tissues and organs [1, 2]. Human-relevant models are created that expand our knowledge of the underlying pathological mechanisms in diseases [3–7]. The pharmaceutical industry and academia use bioprinting with human cells to screen drugs, including for personalized care [5, 7, 8]. Bioprinting promises to bridge the translational gap between pre-clinical findings and successful clinical trials since, often, the drugs that exhibited positive results in animal models fail in clinical settings. Another emerging application of this technology is organ transplantation [9]. Bioprinted organs made using the patient’s own cells will eventually solve the problems of donor shortage and immune rejection [10]. Treatments of burns and other skin injuries are expected to be one of the first clinical applications of bioprinting [3, 4, 6, 11]. The recently passed US Congress ‘FDA Modernization Act 2.0’ encourages using alternatives to animals for drug testing [12]. Bioprinting is such an alternative.

Bioprinting is analogous to the widely used 3D printing technology that manufactures solid structures with pre-defined geometrical and mechanical properties [2] from plastics, ceramics, and metals. Similarly, bioprinting forms gel-like solid biological structures from bioinks that consist of hydrogels, cells, cell media, and cross-linking agents. A wide variety of biological composites have been developed that are tailored to a specific tissue or organ of interest [1]. During the post-printing process, cells undergo modifications, such as differentiation, proliferation, and junction formation. The evolution and maturation of the printed cell-laden constructs are referred to as four-dimensional (4D) bioprinting [13, 14], where the fourth dimension is time. The major challenge related to 4D bioprinting is consistent and reliable delivery of nutrients to and removal of waste products from the bioprinted tissues. Adequate tissue oxygenation is most critical for attaining bioprinting goals. Even transient hypoxia may not only affect cell development but also trigger apoptosis. To overcome the problem of oxygen (O_2_) deficiency in thick (> 1 cm) prints due to underdeveloped vasculature, several strategies have been implemented to supply cells with O_2_ during neovascularization. Chemical (peroxides [15, 16]) and biological photosynthesis (algae [3, 17, 18]) approaches have been utilized toward this goal. Introducing such oxygen-generating materials into bioinks has been evaluated as a solution to the intermittent hypoxia problem [19–21]. Oxygen generation and delivery must be optimized to ensure long-term bioprinted construct progression to the functional tissue or organ. Towards this goal, the 4D bioprinting technology has to be supplemented with 4D oximetry (O_2_ mapping in 3D space and time). Several imaging modalities can be considered for this purpose. Given the tissue size of centimeters, the utility of traditional optical methods is questionable. Light scattering and absorption by biological substances limit 3D optical imaging depth to several millimeters at best. Oxygen-detecting techniques of positron emission tomography (PET) and magnetic resonance imaging (MRI) can be used as they are not limited by penetration depth. However, both modalities have practical limitations in the context of longitudinal 4D oxygen imaging. PET requires the introduction of a radioactive tracer [22]. Long-term exposure of cells to radiation is problematic. Also, the tracer may not be delivered because of underdeveloped vasculature. The latter argument is applied to MRI as well. The indirect hemoglobin-based detection method would only report O_2_ within the bioprinted vasculature [23]. The direct isotope O-17 MRI is prohibitively expensive [24]. It is also restrictive. It would be practically impossible to make algae cells generate O-17 by means of photosynthesis. Functional electron paramagnetic resonance imaging (EPRI) is proposed as a tool that serves the 4D bioprinting needs [25–35]. EPRI is analogous to MRI but detects signals generated by the electron rather than nuclear spins. *In vivo* low-frequency (250—800 MHz) EPR imaging is routinely used in various rodent studies [26, 27, 35]. Spin probes have been developed that report not only oxygen but also a wide range of other biologically significant factors, such as pH, inorganic phosphate, enzymatic activity, and redox status [26, 30, 32, 36,38–48]. These probes can be introduced into the bioinks pre-printing and/or into the bioprinted constructs post-printing. This study, which presents the first integration of 4D bioprinting and 4D molecular imaging, is limited to oximetry.

EPRI application to tissue engineering is relatively novel. A commercial EPR instrument manufactured by O2M Technologies has become available that has been used in studies related to oxygen mapping within engineered tissue models and biomaterials [34, 49, 50]. This instrument is based on the pulsed EPR method that permits imaging of oxygen-sensitive probes with relatively long relaxation times. In this study, we used a different EPR method called rapid scan (RS) [25, 51–57]. RS is a relatively novel technique that is less restrictive with respect to probe relaxation times as compared to pulse EPR. In addition, RS EPR permits the use of a wide range of multi-functional, multi-line electron spin reports of the tissue microenvironment that will be used in the future.

Lithium octa-n-butoxy-phthalocyanine (LiNc-BuO[43]) [52, 58, 59] microcrystals were embedded into bioinks prior to bioprinting to enable longitudinal oxygen imaging. These oxygen-sensing particles are chemically inert and can persist *in vivo* for years. Polydimethylsiloxane-encapsulated LiNc-BuO crystals (OxyChip) have been used in clinical trials [41, 60]. In this study, bioprinting was performed using lab-built bioprinters of extrusion and digital light processing (DLP) types. The DLP technology delivers high resolution and speed. The 3D structure is created by sequential photo-polymerization of 2D slices. Gelatin methacrylate (GelMA [61, 62]) was used as a bioink that cross-links when exposed to violet (405 nm) light. Loading the bioink with an oxygen probe requires adjustments to the existing bioprinting protocols [63].

EPR spectroscopy and imaging experiments revealed an important phenomenon that had not been described in the literature: the photoinitiator used to initiate GelMa cross-linking persists in significant quantities post-printing. When exposed to ambient light, the residual photoinitiator reacts with oxygen, resulting in O_2_ depletion throughout the bioprint. This process may potentially create hypoxic conditions for cells. 4D RS EPR technology is proposed as a means to visualize and quantify this problem. The post-printing maps of the photoinitiator can be acquired indirectly based on the oxygen depletion after light exposure. EPRI is suggested to ensure the complete removal of unwanted residual chemicals post-printing by washing and/or perfusion.

Proof-of-concept 4D oximetry of a bioprinted hollow cylinder model was demonstrated. EPR probes, HEK293T cells, and cell media were incorporated into GelMA hydrogels. Longitudinal oxygen consumption by the cells was measured within the bioprinted model.

## Material and Methods

### Rapid scan EPR imaging

A previously described [25, 26, 52, 54, 55] lab-built rapid scan (RS) EPR instrument operating at ~ 800 MHz was used for imaging. This instrument was originally designed for pre-clinical imaging. A recently developed digital automatic control RS EPR upgrade was utilized for data acquisition [25]. The upgraded imaging system permits uninterrupted data acquisition without the need for manual resonator tuning and coupling. Critical coupling conditions are automatically re-established within tens of milliseconds. Four-dimensional (4D) spectral-spatial images were reconstructed from 3276 projections (RS spectra acquired at varying field gradients) using an algorithm published by Komarov et al. [64]. The maximum magnetic gradient value was 4G/cm. Projections were deconvolved from the corresponding RS signals [53, 56, 65]. The gradient vectors were sampled both in direction and amplitude. All data processing was done using locally written MATLAB software. Reconstructed images were saved as 4D spatial-spectral matrices. EPR spectra were analyzed using MATLAB's *lsqcurvefit* fitting function. The function’s outputs were the integral intensity (proportional to the local concentration of the spin probe) and EPR linewidth. Upon fitting, each 4D image was transformed into two 3D maps for probe distribution and linewidth (LW), respectively. The linewidth maps were converted into oxygen images using a known linear calibration function [66].

Oxygen kinetics with spatial resolution is measured by acquiring a series of 3D O_2_ maps with the time intervals between the images defined by the RS signal averaging time and the number of projections. The temporal resolution for the reported results was approximately 33 min.

### DLP Bioprinter Construction and Software

**Fig. 1:**
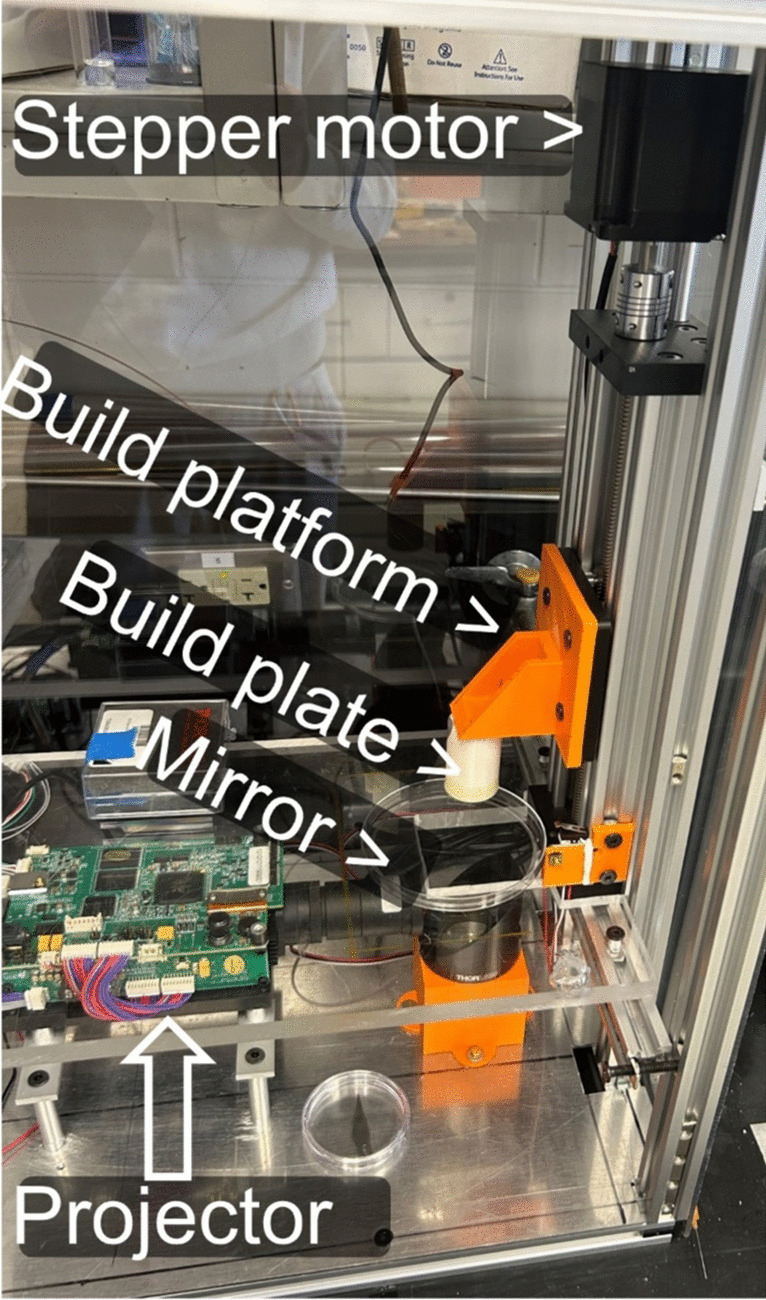
DLP bioprinter.

A digital light processing (DLP) bioprinter was built in the laboratory (see Fig. 1), inspired by the stereolithography tissue engineering (SLATE) design [63]. The key element of the DLP printer is a 405 nm Wintech Pro 4500 projector (Wintech, USA, CA) that generates 2D photomasks according to the uploaded series of bitmap files. The Pro 4500 polymerizes GelMA in a layer-by-layer fashion according to the pre-loaded sequence of 2D bitmap images. Liquid GelMA solution was placed in standard six-well plates. The bottom of each well was coated with polydimethylsiloxane (PDMS) to avoid adhesion of the prints to the wells. PDMS layer thickness was approximately 1 mm. The treated well, which constitutes a bioprinting vat, was irradiated from below using a 45-degree angled mirror (Thorlabs, USA, NJ). The light passes through the treated well and causes polymerization of a thin GelMA layer on the build plate (white cylinder in Fig. 1). This plate is mechanically detachable from a 3D printed platform (orange component in Fig. 1). The platform was vertically translocated using with a standard NEMA 17 Stepper Motor. The motor was controlled by the Arduino Mega 2560 Rev3 (Arduino, Italy) microcontroller, which utilizes a generic Microstep Driver that powers the motor. Synchronously with the plate advancement, Arduino triggers the DLP projector to output a 2D light pattern that polymerizes a layer (~ 100µm) of GelMA. In between the printed layers, the build plate was moved up and down by approximately 10 mm for the purposes of mixing GelMA with EPR probes that tend to precipitate. An aluminum frame was built to house the printer. Polycarbonate sheets were attached to the frame to protect photo-sensitive GelMA from ambient light and contamination.

MATLAB scripts were written to create sequences of 2D bitmap slices from a 3D digital model. The DLP Projector software (DLP® LightCrafter 4500, Texas Instruments, TX, USA) was then used to upload the bitmap files to the projector. A MATLAB Graphical User Interface (GUI) app was developed, permitting printing control. The user could optimize such parameters as layer height, exposure time, and number of patterns/layers. The GUI app communicated with the Arduino through a serial connection.

### GelMA preparations

GelMA was synthesized using previously published methods [67]. Gelatin powder (50 g) derived from porcine skin (Sigma-Aldrich G2500) was dissolved in 500 mL of phosphate-buffered saline solution (PBS, Gibco) at 50 °C while the solution was vigorously stirred until complete gelatin dissolution. Methacrylic anhydride, 30 g (Sigma-Aldrich 276685), was drop-wise added to the solution at 50 °C under stirring for 2 h. The mixture was dialyzed against distilled water, changed daily, using 12–14 kDa cutoff dialysis tubing for 1 week at 50 °C. The final mixture was lyophilized for 1 week to produce a porous white foam that was later stored at ~ -20 °C until further use.

The hydrogel solution used for bioprinting was prepared by dissolving solid GelMA (10% w/v) and lithium phenyl-2,4,6-trimethyl-benzoyl phosphinate (LAP) photoinitiator (0.3% w/v, equivalent to 10 mM) in PBS.

### Optimization of LiNc-BuO-Laden Bioink for Bioprinting

The DLP bioprinter sequentially polymerizes 2D layers of GelMA. Horizontal resolution with each layer (≈ 30 × 60 µm) is defined by the projector specifications. The vertical resolution is established by the build plate displacement (100 µm) and light exposure time (5–8 s per layer). The latter was optimized for the optical properties of the bioink used for printing. Light-absorbing compounds are utilized in bioprinting to avoid polymerization within previously printed layers. A non-toxic food coloring, tartrazine, was used for this purpose [63]. Adjustments of tartrazine concentration were made to compensate for light scattering and absorption by LiNc-BuO crystals. In addition to tartrazine concentration, the timing of light exposure was adjusted to achieve three somewhat antagonistic goals: (i) vertical resolution of ~ 100 µm, (ii) accurate representation of the digital 3D model, (iii) adhesion to the build plate and not to the vat. The latter would tear the print apart. Tartrazine limits cross-linking to a thinner layer and eliminates light scattering in the horizontal plane. It also suppresses cross-linking within GelMA if the concentration is relatively high. Longer time exposure is needed to compensate for the light absorption by tartrazine. However, if exposure times are too long, oxygen is depleted within the PDMS layer, causing GelMA adhesion to the vat, which would break the print. Also, the bioprinting time will become impractically long, especially if cell-laden bioink is used. The bioink used for this DLP 3D bioprinter consists of 10% (w/v) GelMA, 0.3% (w/v) LAP, 2 mg/mL LiNc-BuO EPR probes, and 0.0375% (w/v) tartrazine. Without the LiNc-BuO probe, the concentration of tartrazine is 0.05% (w/v). For bioprinting with HEK293T cells, tartrazine concentration was reduced to 0.0125% to account for the light absorption by the cell media. The time exposure was eight seconds per layer.

### Preparation of LiNc-BuO-loaded GelMA hydrogel

**Fig. 2:**
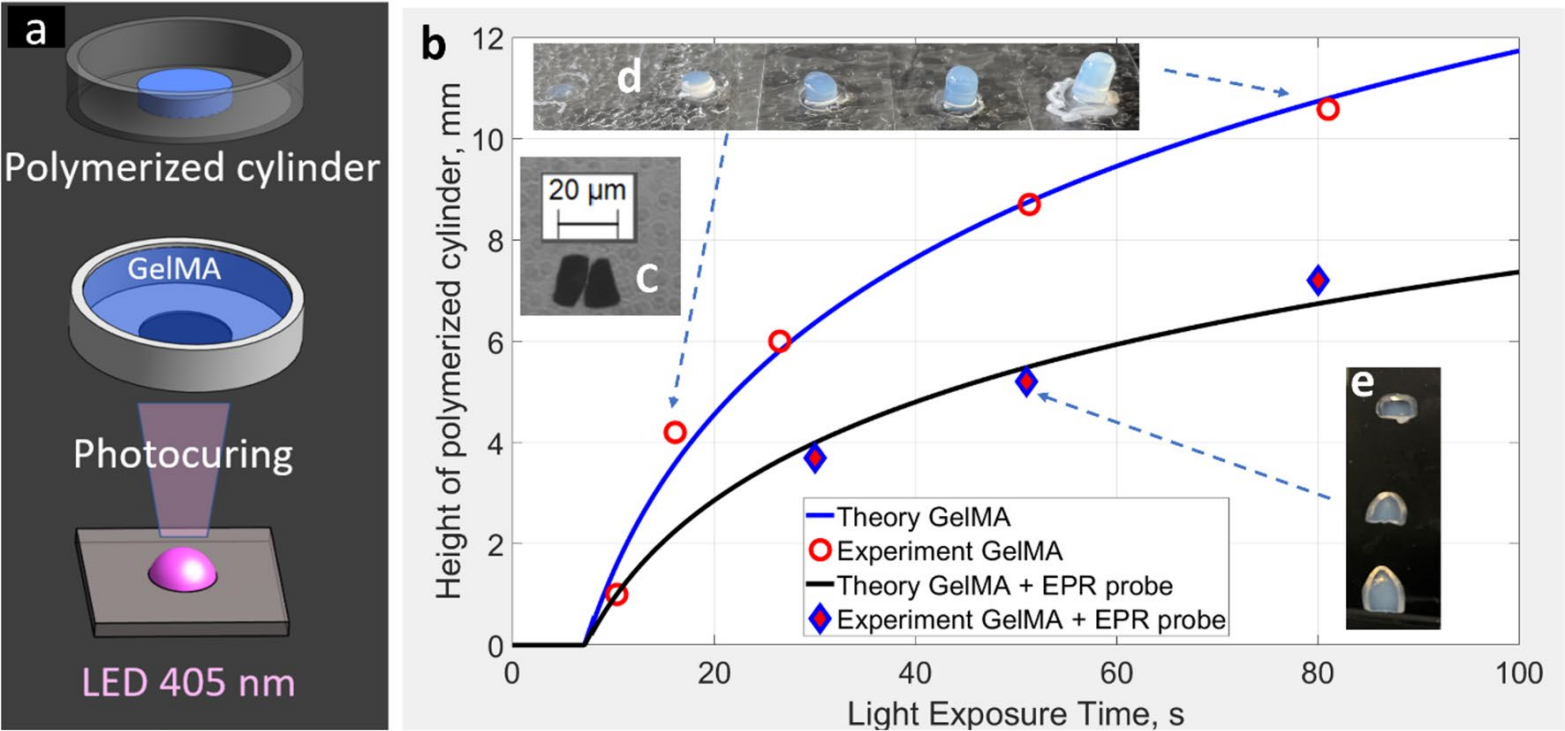
Photopolymerization of GelMA bioink depending on the light exposure time. (**a**) Description of the experimental setup. (**b**) The dependence of photopolymerization front propagation on 405 nm light exposure time. Due to light scattering the heights of polymerized cylinders were relatively lower in the presence of LiNc-BuO probes. Comparison of photographed cylinders with and without the addition of EPR probes shows difference in shapes. LiNc-BuO crystals sharpen the photopolymerization front. Equation (5) was used for data fitting. For the GelMA data (blue curve), t_s_ = 7.2 s and µ = 0.22 mm^−1^. Fitting of LiNc-BuO loaded GelMA was done using the following parameters: t_s_ = 7.2 s and µ = 0.36 mm^−1^. Insert (**c**) Microscopic image of LiNc-BuO particles (two black crystals of ≈10 × 20 µm in size). Insert (**d**) Polymerized GelMA cylinders. Insert (**e**) Polymerized GelMA with the addition of EPR probes.

LiNc-BuO oxygen-sensitive crystals were synthesized from 1,4,8,11,15,18,22,25-octabutoxy-29H,31H-phthalocyanine using a published procedure [68]. The LiNc-BuO probe suspension was prepared using a microfluidic technique. Two syringe pumps (KD Scientific, MA USA) were used to drive a flow of LiNc-BuO (benzene, 1% w/w) and polyvinyl alcohol (PVA in water, 3% w/w) through 100 µm flow chip (Dolomite, UK, part number 3200130). Flow rates of the oil and water phases were set to be equal to 30 and 300 μl/min, correspondingly. The microemulsion was collected in a 3% PVA water solution (200 ml glass cylinder) while the solution was stirred and heated at 95 °C by positioning the container in the water bath. After collecting all droplets, the solution was cured for an additional 30 min and cooled. The suspension was centrifuged at 1000 xg and reconstituted in the 3% PVA water solution, resulting in a solution of LiNc-BuO particles at a concentration of 10 mg/ml. The particles were approximately ~ 10–20 µm in size (see Fig. 2c). Immediately before printing, LiNc-BuO crystals were dispersed in GelMA by gentle stirring.

### Light-emitting diodes (LED)

Two LEDs were used in the experiments, both generating light at 405 nm. The more powerful MTSM405UV-D5120S LED (Marktech Optoelectronics, USA, NY) was utilized for the photopolymerization of GelMA as described in Fig. 2. This LED was supplied with a forward current of 0.44 A, generating approximately 900 mW of radiant flux power. A less powerful LED (~ 100 mW) TCN0MF1A by Sensor Electronic Technology (USA, SC) was used for all other experiments.

### High-Performance Liquid Chromatography (HPLC)

The HPLC device, Waters 2695 (Waters, MA, USA), was equipped with a reverse phase column (4.6 mm * 50 mm). The measurement of LAP concentration was performed using 376 nm light at 45 °C.

### Characterization of LiNc-BuO loaded GelMA for bioprinting

An experimental setup schematically illustrated in Fig. 2a was assembled to evaluate the interaction of 405 nm wavelength light with LiNc-BuO. Liquid GelMA bioink with and without LiNc-BuO probes was placed in a transparent vial. The LiNc-BuO concentration was 1 mg/mL. A light-blocking mask was placed underneath the vial, leaving an exposed circular spot with a diameter of 5 mm. An LED light source was placed below the vial at a distance of 41 mm. The samples were exposed to light for increasing time intervals, forming polymerized cylinders. Unpolymerized GelMA was removed by washing in PBS, and the height of the cylinder was measured. The procedure was repeated for several light exposure time durations, as shown in Fig. 2b.

Experimental data were fitted to a theoretical function derived using the following assumptions. It is well-known that photopolymerization does not start until the excited photoinitiator depletes oxygen, as molecular O_2_ is an inhibitor of the reaction. The concentration of consumed oxygen, O_C_, along the propagation direction can be expressed as

1 $${\mathrm{O}}_{C}=\mathrm{Kexp}\left(-\mu z\right)t;\; z\ge 0,$$

where z is the distance from the vial bottom along the light propagation direction, K is the proportionality coefficient, µ is the attenuation coefficient, and t is the light exposure time. Photopolymerization begins when the initially present oxygen at the ambient concentration of O_A_ is fully consumed. At the vial bottom (z = 0), a time interval t_s_ is required to complete depletion:

2 $${O}_{A}=\mathrm{K }{t}_{s}$$

During this time, no photopolymerization takes place. Substitution of Eq. (2) in Eq. (1) gives:

3 $${\mathrm{O}}_{C}={O}_{A}\mathrm{exp}\left(-\mu z\right)t/{t}_{s} ;\; z\ge 0$$

At the polymerization front, O_C_ = O_A_. As a result, Eq. (3) can be simplified to:

4 $$\mathrm{exp}\left(-\mu z\right) t/{t}_{s} =1$$

for the conditions of polymerization initiation. The cylinder height, H, can be described by the following system of equations:

5 $$\left\{\begin{array}{c}H=0, t\le {t}_{s}\\ \mathrm{H}=\mathrm{ln}\left({t/ t}_{s}\right)/\mu , t>{t}_{s} \end{array}\right. .$$

Equation (5) was used to fit data in Fig. 2b.

### Bioprinting and EPR imaging of a cell-laden GelMA construct

The solid GelMA was prepared as described above. It was dissolved to the concentration of 10% (w/v) in Eagle’s minimal essential medium (Lonza, MA, USA) forming a hydrogel solution. LAP, tartrazine, and LiNc-BuO EPR probes were added to the hydrogel at the concentrations of 0.3% (w/v), 0.0125% (w/v), and 2 mg/mL, respectively. HEK293T cells were acquired from the American Type Culture Collection. The cells were incorporated into the hydrogel at a density of 25,000 cells/mL. A hollow cylinder model (ID = 4 mm, OD = 12 mm, Height = 5 mm) was used for printing (see Fig. 6). The bioprinted cylinder was transferred to EMEM medium for a 24-h incubation at 37 °C and 5% CO_2_ in a human cell incubator (Thermo Scientific Heracel 150i). After incubation, the cell-laden construct was transferred to the bottom of the glass tube, as depicted in Fig. 6a. To ensure a continuous nutrient supply during the imaging, 1 ml of EMEM medium was introduced above the insert. The sample was gently pressed to the tube bottom by inserting a rubber cylinder (yellow ring above the red-colored bioprint at the glass bottom). This insert had a 2 mm hole along its axis to permit an escape path for the cell medium as it was pushed inside the tube (red substance above the yellow ring in Fig. 6a). The tube's upper space was filled up with sterile melted Vaseline that was solidified upon interfacing the liquid medium. The goal of this procedure was to insulate the bioprint from the ambient environment, including oxygen and microorganisms. Blocking influx of O_2_ into the bioprint permitted direct unaltered measurements of its consumption by the cells. A series of 4D spatial-spectral EPR images were acquired over the duration of 400 min, forming a 5D dataset. The data were processed to produce a 4D spatial–temporal image of oxygen kinetics. After data acquisition, the bioprinted construct was placed in an EMEM medium containing 2 μg/mL of collagenase enzyme type A and incubated for 16 h. This resulted in GelMA degradation and release of HEK293T cells in the medium. To assess cell viability, Trypan Blue dye (Corning™), which penetrates and stains dead cell membranes blue, was utilized. Live and dead cells were differentiated using a cytometer (Invitrogen Countess 3). This process provided a measurement of HEK293T cell viability under the experimental conditions.

## Results

### Effect of LiNc-BuO microcrystals on attenuation coefficient of photopolymerized GelMA

The effect of the EPR oxygen probe incorporation into GelMA bioinks on the photopolymerization process with the implication for printability was evaluated. LiNc-BuO microcrystals are visibly black. They absorb and scatter visible light, including in the violet range. As such, the introduction of LiNc-BuO was expected to affect bioink photopolymerization.

Based on the results shown in Fig. 2, 405 nm light attenuation of the media increased by approximately 63% due to the presence of the LiNc-BuO probe. As a result, the height of the cylinders decreased (see Fig. 2b insert **e**) compared to the control GelMA (see Fig. 2b insert **d**) due to the excessive light absorption/scattering by the crystals incorporated in the GelMA bioink.

### Proof-of-concept EPR imaging of oxygen in an acellular bioprinted construct

**Fig. 3:**
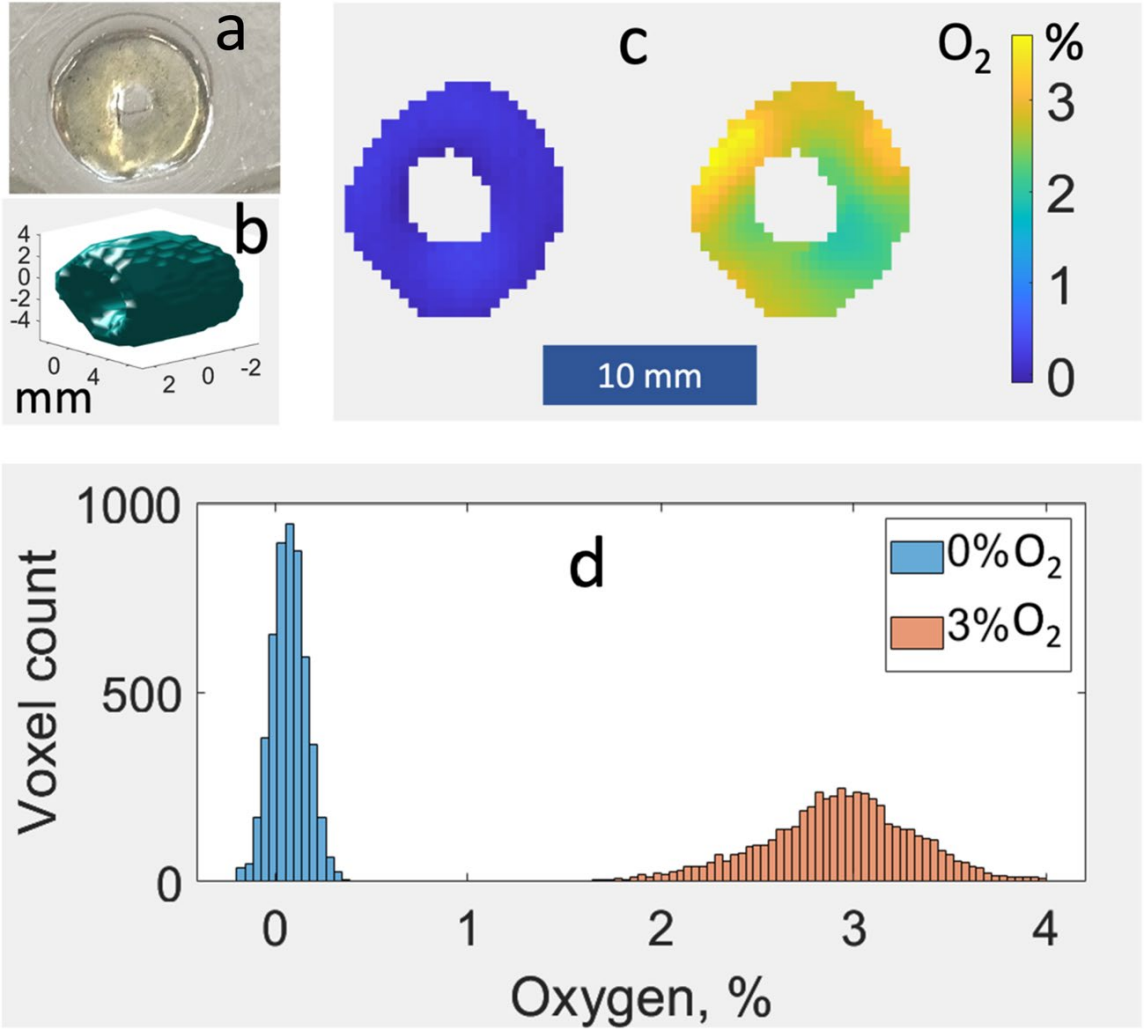
Bioprinted hollow GelMA cylinder with incorporated LiNc-BuO EPR probes. **a** Photo. **b** 3D iso-surface of the reconstructed RS image. (**c**) Central 2D cross-sections of oxygen concentration across the reconstructed images measured at 0% and 3% oxygen perfusion conditions, respectively. **d** Histograms of oxygen distribution in 0% (left) and 3% oxygen (right) images, respectively.

A hollow cylinder (ID = 4 mm, OD = 12 mm, height = 5 mm) was printed using GelMA bioink containing the LiNc-BuO probe (see Fig. 3a). The cylinder was placed in a glass tube containing PBS buffer solution and imaged. A 3% O_2_ and 97% nitrogen (N_2_) gas mixture was bubbled into the liquid covering the cylinder. RS spectra of decreasing linewidth were observed as oxygen concentration was declining from the ambient value (~ 21%). When the linewidth remained unchanged, indicating 3% of O_2_ throughout the sample, a set of projections was measured as described above. After the acquisition of data, the sample was exposed to 405 nm light with the goal of depleting oxygen by its reaction with the residual photoinitiator. Then, the PBS solution covering the sample was continuously bubbled with 100% N_2_ to prevent deoxygenation. Projections were acquired. Reconstructed images corresponding to 0% and 3% oxygen are shown in Fig. 3c on the left and right, respectively. 3D EPR probe distribution is presented in Fig. 3b. Oxygen histograms for 0% and 3% cases are shown in Fig. 3d.

### Oxygen depletion by residual photoinitiator may induce hypoxic conditions

RS spectroscopy and imaging experiments revealed that exposure of photopolymerized GelMA to ambient light caused oxygen depletion within the bioprinted construct. This phenomenon can be explained by the reaction of the residual photoinitiator with O_2_ under light exposure [69]. LAP absorbs light in the range from ultraviolet to ~ 410 nm (violet). As a result, the photoinitiator that has not been consumed during the printing process continues to react with oxygen under ambient light. This is problematic, as photoinitiators may create hypoxic conditions within a bioprinted construct. No description of this potential drawback has been found in the bioprinting literature that can be attributed to the lack of oxygen imaging instrumentation. A study was conducted to evaluate the severity of the problem as well as to suggest mitigating approaches with the goal of estimating the diffusion coefficient of LAP within photopolymerized GelMA and to evaluate the time needed to remove the residual photoinitiator by washing/perfusion. Toward this goal, a disk (38 mm in diameter, 1.7 mm in height) was photopolymerized using the 405 nm LED for a duration of 5 min from 144 mm distance to ensure homogeneous light exposure. The net energy density (~ 5 W/cm^2^) deposited on the disk surface was approximately 35% of that used for bioprinting, with no food coloring added. After the LED light exposure, the photopolymerized disk was digested by the introduction of the collagenase-A enzyme. The supernatant solution was analyzed using HPLC. The measured residual photoinitiator concentration was 4.4 mM, which is comparable to the initial concentration of 10 mM (equivalent to 0.3% (w/v)). This amount is still substantially larger compared to the estimated 0.2 mM of dissolved oxygen at the ambient conditions. In principle, the residual photoinitiator could be removed by extended photocuring. However, this approach would be problematic for several reasons. First, prolonged light exposure may be toxic to the cells. Second, photocuring changes the physical structure of photopolymerized GelMA, causing its shrinkage. Third, light strongly decays when penetrating inside thick bioprints, so the effect would be spatially inhomogeneous. Instead of photocuring, incubation/perfusion of the bioprinted construct with a buffer containing all needed nutrients should be utilized to remove the residual photoinitiator.

**Fig. 4:**
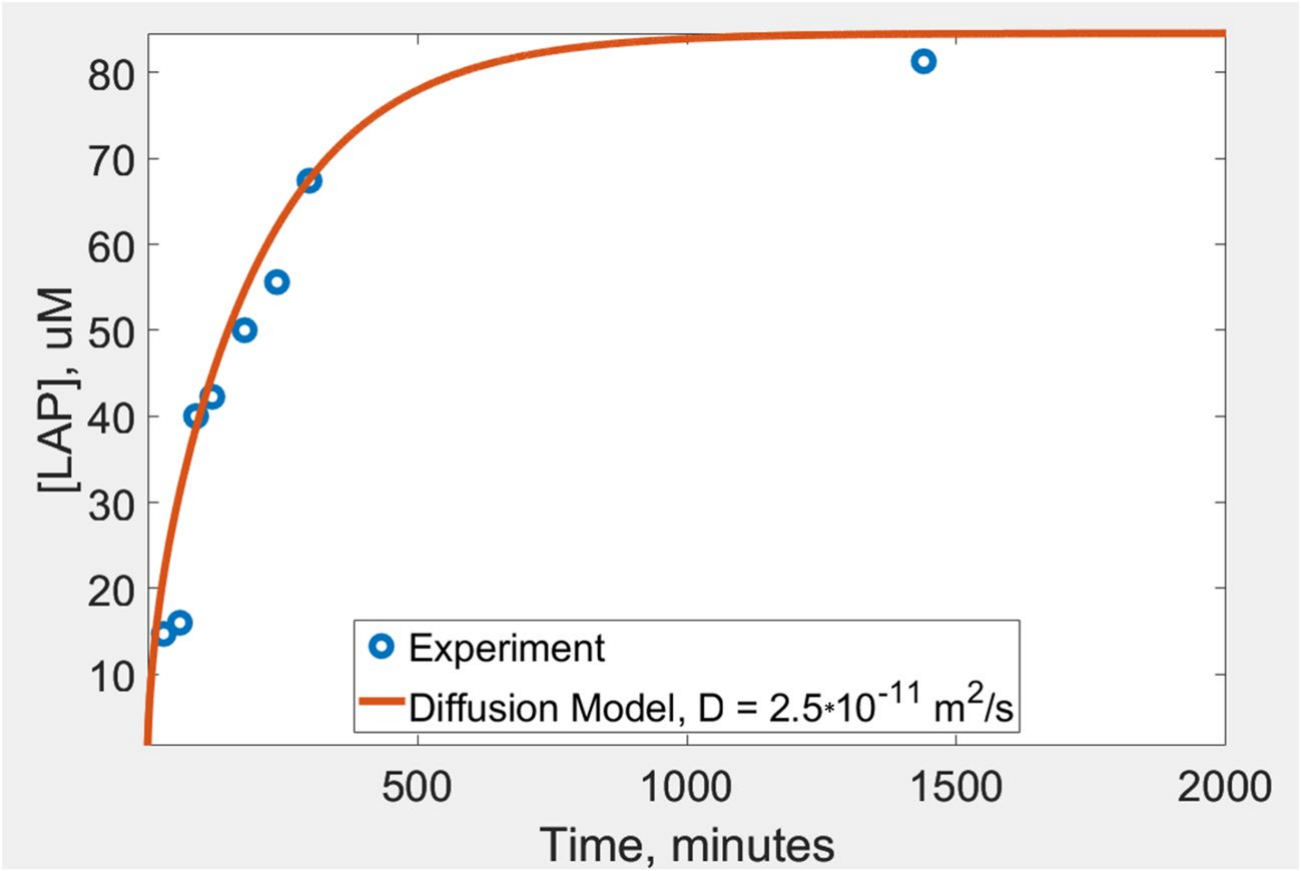
Experimental sampling (blue circles) and modeling (red curve) of LAP concentration in the 100 mL PBS flask containing GelMA disk.

The LAP diffusion coefficient was evaluated using the described above GelMA disk sample. The disk was placed in a 100 mL container filled with PBS. The LAP (initial concentration of 4.4 mM) diffused out of the disk and into the container. The experiment was conducted in the dark. The solution in the container was sampled and measured using the HPLC. The results are shown in Fig. 4. The mathematical modeling (theoretical curve, red) was done in MATLAB by solving a one-dimensional diffusion equation with the following boundary conditions: [LAP] at the interfaces of GelMA is equal to that of [LAP] in the PBS solution. The photoinitiator concentration within the flask volume was considered to be homogeneous at all times. Experimentally, this condition was ensured by gentle stirring.

**Fig. 5:**
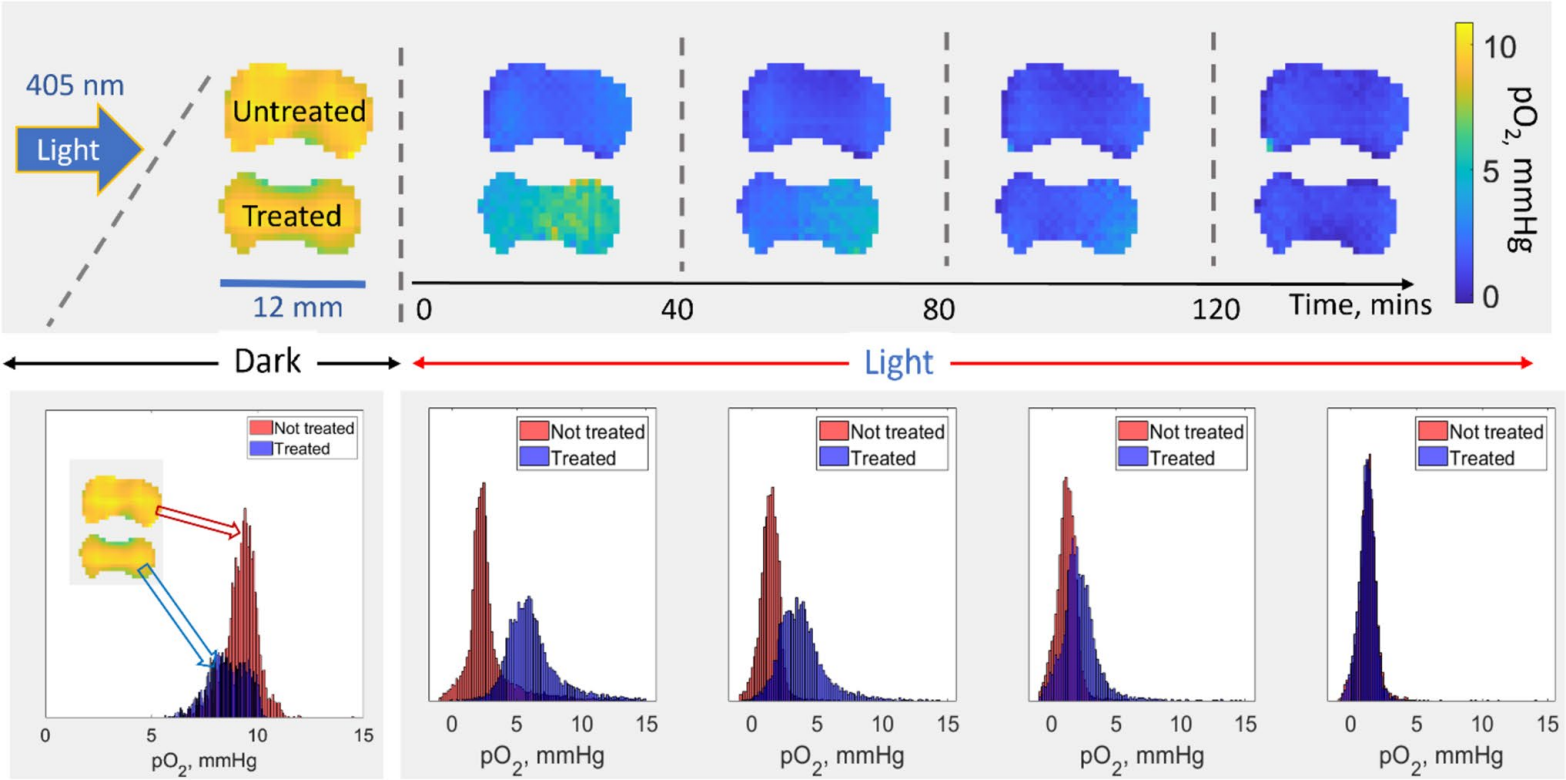
Oxygen depletion in polymerized GelMA/LiNc-BuO samples under light exposure. The top group of image cross-sections shows pO_2_ changes in treated (PBS perfused) and untreated samples over time. The lower panel demonstrates corresponding histograms of pO_2_ distribution within the entire images.

The final measured concentration of the photoinitiator was close to the expected value calculated as 4.4 mM *2/102 = 86.3 µM, suggesting that LAP can be almost completely removed from a relatively thin (1.7 mm) GelMA layer by one day-long incubation. LAP depletion from a thicker solid construct would take an increasingly longer time, which can be evaluated numerically. RS imaging is proposed as a tool to indirectly detect post-incubation LAP presence. A proof-of-concept experiment was done using two photopolymerized GelMA models containing LiNc-BuO probes. One of the constructs was perfused with PBS over a 10-day period. Both samples were purged with nitrogen to lower the oxygen partial pressure to approximately ten mmHg for the purpose of improving sensitivity (EPR spectrum peak amplitude reduces proportionally to the linewidth). Both samples were exposed to continuous 405 nm light and imaged over approximately three hours. Oxygen kinetics of the untreated (top) and PSB-washed (bottom) samples are shown in Fig. 5. Partial pressure of oxygen (pO_2_) dropped almost instantaneously after light exposure. It took a substantially longer time to deplete oxygen in the PBS-incubated sample. LAP concentration in the washed sample was estimated to be ~ 1 mM, which is still large compared to the concentration of dissolved oxygen. This explains oxygen depletion in the perfused sample, although on a much longer time scale.

Control experiments were performed to evaluate the results shown in Fig. 5. An oxygen consumption rate of 0.001% per minute was measured in the PBS buffer in the absence of LAP and GelMA under 405 nm light exposure. The addition of GelMA slightly increased the rate to 0.0035% per minute. The introduction of LAP into PBS resulted in a ≈5000-fold increase in the reaction rate (5.1% per minute). In these experiments, the 405 nm LED source was placed in close proximity to a glass vial (OD = 15 mm; ID = 13 mm) containing the measured samples. A fiber optic oxygen probe IMP-PST7-02-L0,2-LIC0-BJF3-TF-OIW (Presence, Germany) was placed in the tube together with the temperature sensor. The position of the LED with respect to the tube remained unchanged for all three measurements.

### EPR imaging of oxygen consumption within cell-laden bioprinted cylinder

**Fig. 6.**
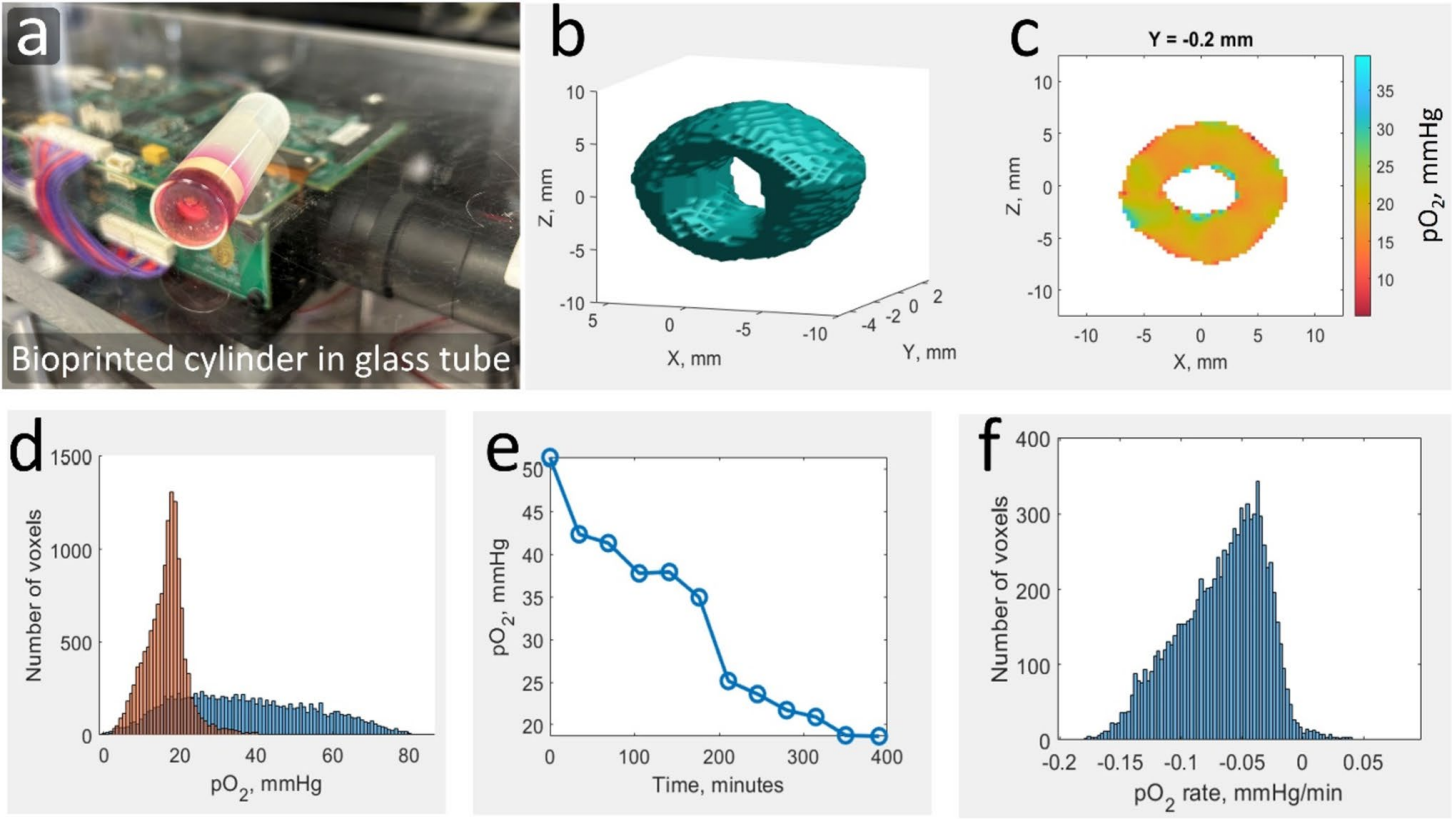
Oxygen imaging of bioprinted cells-laden GelMA constructs. (**a**) Bioprinted GelMA cylinder containing HEK293T cells, fixed in air-tight glass tube while incubated with the EMEM medium. (**b**) surface model of reconstructed EPR image (**c**) 2D cross-section of the latest 3D image at y = -0.2 mm position. Color bar shows pO_2_ in mmHg. (**d**) histogram of first and last image showing distribution of oxygen partial pressure across all voxels in the image. (**e**) representative oxygen partial pressure at one voxel across time. (**f**) histogram of oxygen consumption rate calculated at each voxel.

Oxygen consumption by HEK293T cells within the bioprinted hollow cylinder has been measured using EPRI. The results are shown in Fig. 6. As described in the Materials and Methods section, the sample was placed in the glass tube and sealed off. The imaging experiment was initiated when the level of oxygen reduced from the ambient value to ~ 50 mmHg. The reason for this is a steep linear dependence of LiNc-BuO linewidth on the oxygen partial pressure (pO_2_). This dependence permits pO_2_ measurements with high accuracy. The downside is that LW becomes broad (2 G) at near ambient oxygen pressure (160 mmHg), reducing the spatial resolution of the images to 5 mm when using a 4G/cm maximum gradient. The resolution was approximately 1.6 mm and 0.6 mm at 50 mmHg and 20 mmHg, respectively. The 3D probe distribution map shown in Fig. 6b was computed using the last and best-resolved 3D map out of the total twelve. A 2D cross-section of this image at y = -0.2 mm is presented in Fig. 6c. Figure 6e shows oxygen depletion due to the cellular metabolism at a given voxel. The histogram for the consumption rates for all voxels is presented in Fig. 6f.

Data acquisition was terminated after the average oxygen level dropped to ~ 20 mmHg with the purpose of evaluating cell viability post-imaging. Prolonged hypoxia could have damaged the cells. Cell viability measurement results showed that 30% of cells survived both the bioprinting process and imaging.

## Discussion

Longitudinal 3D oximetry is essential for the development of 4D bioprinting technology. Consistent delivery of oxygen in adequate quantities to the cells is required to ensure the reliability of drug testing. Even transient hypoxia may skew the results. Optical imaging methods exist that can measure oxygen. However, they fail when it comes to 3D mapping of opaque and inhomogeneous substances that are relatively thick (> 1 cm). Light has a limited penetration depth due to its absorption and scattering in biological tissues. In comparison, magnetic resonance methods, such as EPR, are not severely restricted by the penetration depth. RS EPR is proposed as the method of choice to address the need for noninvasive, quantitative, and longitudinal functional imaging of bioprinted models at a low/safe magnetic field. This novel technology permits fast, high-sensitivity acquisition of multidimensional imaging data. A wide range of functional electron spin probes reporting O_2_, pH, enzymatic activity, viscosity, inorganic phosphate, and more have been developed that can be imaged using RS EPR. The availability of existing and emerging functional reporters opens up a wide range of opportunities for biomedical EPR. Bioprinting will greatly benefit from imaging of oxygen and other factors of tissue microenvironment. This study represents the initial steps toward the integration of 4D bioprinting with 4D spatial–temporal EPR imaging.

EPR methodology requires an introduction of exogenic probes. There are two kinds of probes used for oximetry: solid particulate and water-soluble molecules. The solid particles (lithium phthalocyanines [70], India ink [71], Gloxy [72], *etc*.) are persistent, nontoxic, easily implantable, and observable over months in biological samples. They show high sensitivity to oxygen [73]. However, they cannot be noninvasively removed from the tissue. This may be problematic when it comes to organ transplantation. The second type of probes are water-soluble molecules characterized by lower oxygen sensitivity. Triarylmethyl radicals (trityls) are most suited for high-resolution imaging as they can produce narrow EPR spectra with LW lower than 100 mG [40, 74]. Trityls are commonly used for pre-clinical imaging. For bioprinting applications, re-introduction of the probe may be required. It will have to be added to the perfusing media. Otherwise, trityl probes will be washed out of the bioprint.

LiNc-BuO microcrystals were incorporated in bioinks for this study. This probe has been used for preclinical and clinical EPR oximetry [75, 76]. LiNc-BuO crystal suspension in a water-based solution can be prepared using shakers and/or sonication techniques [77–79]. However, the microparticles’ broad size distribution (due to the particle’s aggregation) and relatively low concentration of LiNc-BuO crystals in water suspension make them challenging to use in imaging applications. Therefore, the emulsification technique was implemented here to prepare the LiNc-BuO probe suspension in water media. The microfluidic technique has found widespread applications in the manipulation of microemulsions and the preparation of microparticles. This technique allows for the preparation of highly uniform-sized emulsions and particles. The microparticle's diameter can be controlled by adjusting the flow rates of the dispersible (oil) phase and the continuous (water) phase.

Studies were conducted to evaluate the compatibility of LiNc-BuO microcrystals with the photoinitiated cross-linking process in GelMA bioink, one of the major hydrogels used for bioprinting. Figure 2 demonstrates that crystalline EPR probes expectedly absorb and scatter light. Surprisingly, the scattering primarily occurs along the z-direction of light propagation, as evident from the pointed shapes in the inserts in Fig. 2, not affecting resolution in the xy-plane. It is in this plane that 2D splices are formed during DLP printing. The introduction of LiNc-BuO requires fine-tuning of bioink optical properties. The concentration of light-absorbing additives (tartrazine in this study) has to be reduced. Both compounds (tartrazine and LiNc-BuO) attenuate light. Optimization of the bioink light absorbance is needed to control z-resolution. Such optimization was performed to achieve 100 µm resolution for DLP bioprinting of several models, including a hollow cylinder shown in Fig. 3.

A proof-of-concept imaging was conducted to demonstrate EPR probe responsiveness to O_2_ throughout the print. To efficiently deplete oxygen in the transition from 3 to 0%, the cylinder was illuminated using the 405 nm LED source. Here, a phenomenon of ‘the residual photoinitiator’ was utilized. Relatively large quantities of LAP remain in the bioprints that continue reacting with oxygen under ambient light. The residual photoinitiator has the potential to cause long-term inadvertent hypoxia in bioprinted constructs. Studies were conducted to investigate this undesirable phenomenon unreported by the bioprinting community due to the lack of imaging capabilities. Oximetric RS EPR imaging is suggested as the means to evaluate the oxygen-depleting capacity of the residual photoinitiator in bioprinted constructs. A brief light exposure would induce observable changes in oxygen distribution. The residual photoinitiator (LAP in this study) is proposed to be gradually removed by perfusion of media containing oxygen and nutrients through bioprinted vasculature. The removal rate would depend on the distance to the closest vasculature channel and the diffusion coefficient of LAP in GelMA. The latter was experimentally evaluated using HPLC (see Fig. 4) and MATLAB modeling. LAP removal via fully developed vasculature would be relatively fast as the average distance between the capillary vessels in the perfused tissue is ~ 200 µm. In practice, bioprint maturation takes time, during which blood capillaries are formed. LAP depletion will accelerate during this time. It would be possible to model the LAP removal process numerically using the estimated diffusion coefficient, 3D topology, the rates of perfusion, and the concentration of the residual photoinitiator. The latter was measured to reduce from 10 mM to 4.4 mM within a disk exposed to light for 5 min. These values are much larger compared to the oxygen concentration in bioink (≈ 0.2 mM) at the ambient conditions. It follows that post-printing photocuring may not be practical in solving the residual photoinitiator problem. Long-term light exposure is problematic in the application to bioprinting: (i) prolonged hypoxia; (ii) excessive cross-linking would cause shrinking of the bioprint; (iii) cytotoxicity due to the generation of reactive oxygen species. Perfusion of bioprints while temporarily blocking part of the optical spectrum that excites photoinitiator remains the only universal practical solution to this problem. A permanent light blocking may be practical for some applications where bioprinted constructs remain in a controlled condition, such as in a laboratory setting. This will not be the case for clinical bioprinting applications. There is also a toxicity problem related to LAP and similar compounds (generated free radicals) that needs to be resolved. The capacity for the residual photoinitiator to deplete oxygen upon perfusion can be estimated using RS EPR imaging (see Fig. 5).

The novelty of the results reported here is the incorporation of EPR oxygen probes into the bioink that permitted longitudinal oxygen imaging. Proof-of-concept experiments were conducted using a simple hollow cylinder model with the intention of mimicking a blood vessel. Relatively low (30%) cell viability can be ascribed to the formation of free radicals initiated by the intense violet light [80] during bioprinting. In addition, photo-crosslinking requires the creation of an oxygen-free layer as O_2_ reacts with free radicals and terminates polymerization. Intermittent hypoxia may also contribute to the cell death. In principle, rapid scan EPR can be used to image the formation of an O_2_-free layer and the kinetics of its dissipation to optimize the bioprinting process. A novel radical-free photopolymerization approach was recently proposed that resolves the phototoxicity problem [81]. Extrusion bioprinting is another solution to the problem of radical formation. However, this method causes cell damage due to shear stress [82]. Independent of the printing technology, 4D EPRI can be used to measure oxygen consumption rates within the bioprint and indirectly assess local cell density and viability with spatial resolution.

## Conclusions and Future Directions

4D bioprinting is an exponentially growing field of biomedical engineering that promises fundamental change to healthcare via enabling personalized treatments and, ultimately, organ transplantation. Printing and long-term viability of thick (> 1 cm) biological constructs are the major challenges of this technology. Robust delivery of oxygen/nutrients and waste removal are impeded due to underdeveloped vasculature. There is a need for a 4D imaging technology that would noninvasively monitor tissue microenvironment to ensure adequate oxygenation, optimum pH, and proper quantities of other important biochemical markers. RS EPR imaging has the propensity to become such a tool. This manuscript focuses on oximetric measurements in photopolymerized GelMA, a commonly used hydrogel for bioprinting. Future studies will expand to molecular imaging using other types of EPR probes introduced into a wider range of bioinks. Complex vascularized/perfused tissue models with the inclusion of several cell types will be bioprinted and investigated using longitudinal multi-functional molecular imaging.

## Data Availability

Data presented in this manuscript can be provided upon request.
